# Human Health, Environmental Quality and Governance Quality: Novel Findings and Implications From Human Health Perspective

**DOI:** 10.3389/fpubh.2022.890741

**Published:** 2022-06-24

**Authors:** Liqin Zhang, Yuping Yang, Yesong Lin, Huangxin Chen

**Affiliations:** ^1^School of Economics, Fujian Normal University, Fuzhou, China; ^2^Fuzhou Lianjiang Ecological Environment Bureau, Fuzhou, China

**Keywords:** human health, governance quality, environmental quality, indoor and outdoor temperature, health implications

## Abstract

Human health and wellbeing are intimately linked to the state of the environment. The current study emphasizes the role of environmental quality, government policies, and human health. This paper provides a detailed literature review of existing findings regarding our key variables of interest. The results argue that the implications of poor government policies and environmental pollution for rising economic development have led to poor environmental quality and health issues for humans. Based on earlier investigations, the present study reviewed the state-of-the-art review and determined innovative insights for outdoor and indoor environment difficulties. This study provides a detailed review of human health, environmental quality, and governance quality. In addition, the study conducts an empirical analysis using the annual data of low-income countries from 1996 to 2020. Government actions and health systems must be modified immediately to address these rising concerns successfully. The report offers policy recommendations for addressing health, governance, and environmental change mitigation issues, all of which are directly or indirectly related to the study. This article presents an overview of environmental change's health impacts and explores how health hazards may be reduced or eliminated through effective adaptation strategies.

## Introduction

Health is inextricably linked to climate change. Global climate variations have influenced human health. A clean environment is essential for human health and wellbeing. On the other hand, unrestrained and uncontrolled development contributes to environmental health issues because it overexploits the natural environment and its resources. Environmental health challenges spread across country borders, making them worldwide issues. The consequences can be immediate and chronic, including water-borne infections caused by inadequate sanitation or skin cancer caused by exposure to arsenic in groundwater or excessive UV radiation (due to depletion of the stratospheric ozone layer). Because of technological activity, environmental degradation has started in emerging nations and the growth of air emission sources such as automobile vehicles (1–5). Most of these casualties (3.3 million and 2.6 million, respectively) are found in Asia, in which indoor air pollution kills 4.3 million individuals and air temperature pollutants kill 3.7 million. As a result, it is very important to identify the problem, notably its hazardous consequences on human health, and provide policy suggestions for saving the environment and human health.

Growing environmental pollution has caused major concern to population lives since the liberalization and deregulation, in tandem with rapid economic expansion (6–12). Wildfires in Australia and California, China's worst floods in decades, the first-ever heatwave in Antarctica with rising temperatures above 20 °C, microplastic discovered in Antarctic ice, and crop destruction by locusts swarming across parts of Africa, the Middle East, and Asia occurred in the year 2020 alone. Habitat loss is another important environmental problem, and it is rapidly being caused by land clearance for agriculture cash crops, e.g., fruits and vegetables, sugarcane, and palm oil, making agriculture the largest driver of deforestation (13). Poor air quality is a third important environmental catastrophe. According to world health organization (WHO) estimates, 4.2 to 7 million people die each year due to air pollution worldwide (14). According to research by the EU's environmental protection agency, roughly 400,000 people died in the EU in 2012. According to UNICEF estimates, 258,000 people died in Africa in 2017 because of air pollution. Following the COVID-19 pandemic, researchers have focused their attention on the impact of air pollution on viral movement (15–20). Recent studies have also found a link between air pollution and COVID-19 pandemic related deaths and between air pollutants and human disease transmission (15, 18). According to researchers, if quick legislative and regulatory steps at the global and national levels are not implemented, five new pandemic illnesses will arise annually (21). They focus on global politicians, whose recommendations on long-term economic developments are crucial and can significantly prevent additional environmental harm.

Good governance, also referred to as green governance, gained some traction following the Stockholm United Nations Conference on Human Development in 1972, establishing the United Nations Environment Programme (22). As a result, policies on environmental governance at the international and national levels have become increasingly popular. Furthermore, we are interested in the influence that formal state-imposed laws and regulations may have on this research's economic activity and environmental quality. In addition, a lack of enforcement or the evasion of current legislation (for example, by outsourcing) may lead the activity to be illegal to shift from the formal (more regulated) sector to the informal (less regulated) sector. As a result, our second indicator of registration and licensing links to more direct governance indicators, such as corporate governance, economic stability, government effectiveness, and voice and accountability.

The article examines the link between the administration and ecological sustainability and perhaps develops policy recommendations for states to preserve and enhance environmental consequences. The importance of this study is shown by the positive (or negative) effects of excellent (or bad) policy in terms of environmental quality. Non-compliance must be punished under better-governed policies and processes. Multiple voluntary environmental contracts have frequently resulted in suboptimal effects on the environment. As a result, the impact of governance on environmental consequences must be investigated. The study contributes to understanding the impact of governance on environmental quality in various areas.

This review has tried to summarize the environmental pollution, governance problems, and appropriate management. As a result, health, and environmental experts, notably policymakers, emergency physicians, and other clinicians concerned with air quality and catastrophes, will find it beneficial. This study also examines the origins of pollutants in the environment, the diseases caused by environmental pollutants and proposes feasible solutions that may benefit environmental legislators and decision-makers. The following is how the rest of the article is organized: Section Interconnected Literature Review explores the interconnected literature review about how good and bad governance can affect environmental quality and human health? Section Environmental Quality vs. Human Health examines the quality of the outdoor and indoor environments and how they affect human health. Section Empirical Results explains that climate change and health is emerging challenge for the world. Section Discussion On Climate Change, Governance, and Public Health will discuss the policy implications for human health and better environmental outcomes.

## Interconnected Literature Review

The new civilized world's primary concern is environmental quality, and it has a big toxic impact on people's health and society. It has a wide range of pollution sources, including automobiles, responsible for the majority of pollution. The six principal contaminants are particle pollution, ground-level ozone, carbon monoxide, Sulfur oxides, oxides of nitrogen, and lead. People are harmed by toxicants floating in the air, including respiratory and cardiovascular ailments, neuropsychiatric issues, eye irritation, skin diseases, and long-term chronic diseases like cancer (23). Pollution comes in many forms, from single cigarettes to natural disasters such as volcanic activity and enormous emissions from automobiles (24). The long-term impacts of environmental damage on the onset of diseases such as lung problems and inflammatory disorders, cardiovascular disorders, and cancer are well documented (25), but air pollution is linked to the deaths of millions of people worldwide every year (26). Another study discovered a link between male infertility and air pollutants (27).

Although numerous studies have identified intriguing correlations of health disparities, environmental and population health studies are still mainly distinct domains. As a result, little is known about the risk attributable to social and environmental variables or how these hazards may interact to generate synergistically or ongoing costs to the health of the populations. There is a need to make environmental policies that lift the environmental cleaning for better human health and a healthy society.

The problem of environmental racism is a specific form of environmental injustice. “Environmental racism” is defined as “racial discrimination in environmental policymaking, regulation and enforcement agencies, the purposeful attacking of communities of color for toxic waste facilities, the official sanctioning of the life-threatening presence of poisons and pollutants in our communities, and the history of excluding people of color from the governance of ecology movements.” Because of economic disempowerment and policies, no community is forced to bear a disproportionate share of the negative human health and the environment consequences of pollution or environmental effects due to industrial, state, city, and commercial projects or the implementation of federal, state, local, and tribal programmers. Table 1 summarizes the studies on government policies and their environmental impact and human and animal health.

**Table 1:** Literature summary of governance policies impacted on the environment.

**Authors**	**Analysis**	**Results**
Fang et al. (28)	New growth models	Environmental protection and environmental transition encounter various, unpredictable, and cross-scale difficulties.
Rehman et al. (29)	Theoretical and empirical analysis	The policy approach has a high priority and is well-understood.
Shang and Xu (30)	Theoretical and database assessment	Make the distinction between environmental management and environmental governance.
Payne and Apergis (31)	Analysis of stochastic and group convergence from per capita greenhouse gas emissions	Address environmental governance as a regulatory framework, institution, and organization system that allows public officials to explain environmental concerns and influence environmental results.
Tarazkar et al. (32)	Kids and families of working age have a favorable influence on the environment.	Government environmental laws may have an “intellectual compensating effect,” encouraging more offshore manufacturing of greener technology to enhance the quality of the environment.
Aslan and Altinoz (33)	Theoretical paper	The federal government is involved in environmental preservation efforts.
Li et al. (34)	Economic data	Local authority officers' influence, conduct, and governing experiences impact its economy and environmental quality.
Teng et al. (35)	Based on pollution data	The amount of money spent by the government on the protection of the environment has a substantial impact on pollution control.
Wang et al. (36)		Local authorities, at various levels, play a critical role in attaining the protection of environmental goals.
Wu et al. (37)		Examine the influence of government management and citizen engagement on local environmental quality.
Yu et al. (38)		Demands from the public are favorable to a modest decrease in energy usage and improved environmental governance efficiency.

An increasing number of individuals and organizations are becoming concerned about environmental protection. According to the experience of industrialized nations such as the United States and the European Union, a comprehensive environmental public administration comprises the government and the public community (39). In several other nations, such as China, public engagement has lately emerged as an emergent topic for environmental governance. The Chinese government has already defended the public's legitimate environmental rights by establishing environmental protection hotlines and mailboxes.

The relationship between income, energy, and carbon emissions has received a lot of attention in the literature, but most empirical research hasn't looked at the influence of the real estate market on their models. Non-renewable sources of energy, such as oil, natural gas, and coal, have been shown to increase Emissions of CO_2_ in past studies (40–43). Renewable energy, on the other hand, can help to reduce pollution. According to another study, renewable energy use adds considerably to CO_2_ emissions, whereas real income promotes short-term and medium-haul environmental destruction (44, 45). This study aims to add to the empirical literature by looking at the impact of insurance market development on environmental degradation.

Another study found that economic development and renewable and non-renewable energy usage have a beneficial impact on CO_2_ emissions (46). Additionally, this research shows that the actual estate market in Turkey has a detrimental impact on reducing carbon emissions. Another study uses estimating approaches to disclose UAE officials' major findings and policy recommendations (47). This study also includes a substantial amount of empirical evidence and financial development (48, 49).

## Environmental Quality vs. Human Health

**Figure 1:**
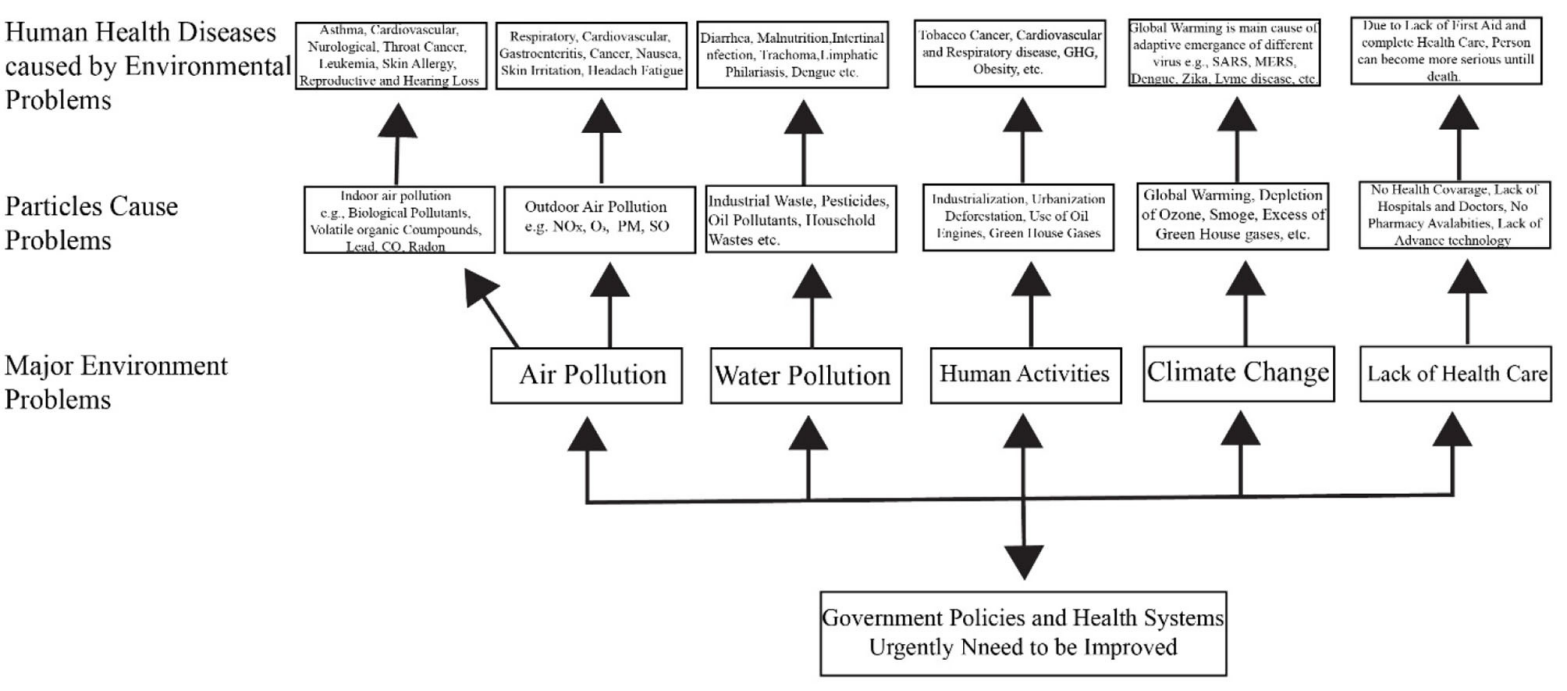
Some major environmental problems and their pollutants cause major disorders in human health.

External air temperature, daily temperature range (DTR), temperature extremes, and other factors influence the external environment (both in and out of house and offices). According to studies on the relationships between temperature and human health, the effect of ambient temperature on public health produces cardiovascular problems (Figure 1). Additionally, because the modern lifestyle necessitates spending a lot of time indoors (at home and work), the indoor environment is critical to human health. Despite this, studies on the impact of the interior environment on human health are rare. According to certain studies, an undesirable interior atmosphere might cause serious health problems in youngsters. Cardiovascular and pulmonary death rates were higher than other ailments: digestive system problems, infectious infections, and premature birth. The impacts of indoor and outdoor settings on human health are summarized in Table 2.

**Table 2:** Literature summary on the outdoor and indoor environment, their causes, and health issues.

**Authors**	**Environment type**	**Causes**	**Disease**
Ban et al. (50), Ge et al. (51), Cui et al. (52), and Li et al. (6)	Outdoor	Changes in the ambient temperature because of climate change	Heart disease (CVD) is a condition that affects changes in surrounding temperature and is linked to morbidity and death.
Ma et al. (53) and Liu et al. (54)	Outdoor	Temperatures in the ambient air that are too hot	Diseases of the lungs
Wang et al. (55) and Li et al. (56)	Outdoor	Environmental factors (PM)	Diseases of the gastrointestinal tract
Li et al. (57)	Outdoor	Environment and temperature exposure	Deaths due to endocrinology and metabolic disturbances
Yang et al. (58)	Outdoor	Environment and temperature exposure	Deaths caused by diabetes
Yang et al. (58)	Outdoor	Environment and temperature exposure	Colic of the kidneys
Liu et al. (59)	Outdoor	Environment and temperature exposure	Renal syndrome and hemorrhagic fever
Li et al. (60), Duan et al. (61), Wang et al. (62), and Zhao et al. (63)	Outdoor	Environmental factors	Viruses and other infectious disorders
Wang et al. (64)	Outdoor	Ambient air temperature	Infectious diarrhea
Chen et al. (65), Jiang et al. (66), Wang et al. (67), and Xu et al. (68)	Outdoor	Ambient air temperature	Hand, foot, and mouth disease (HFMD)
Liang et al. (69), Liang et al. (70), Ding et al. (71), and Guo et al. (72)	Outdoor	Cold temperature	Preterm birth
Fan et al. (73)	Indoor	Meteorological conditions (i.e., air temperature and relative humidity)	Children with asthma and respiratory illnesses
Fan et al. (73)	Indoor	The pollutant in the air	Signs inside and in the lungs
Zhang et al. (74), Lu et al. (75), and Qian et al. (76)	Indoor	Temperature and emission of toxic pollutants in the air	Pediatric allergies and asthma are linked to indoor mold and moisture.
Deng et al. (77) and Liu et al. (78)	Indoor	Indoor variations in temperature	Renovations, cooking, pet care, and human health are considered.
Fan et al. (73)	Indoor	The ambient temperature is frigid in the winter, and the relative humidity is low; the surface temperature is hot in the summer, and the humidity is high.	Effect the children's health
Qian et al. (76)	Indoor	Dry and humid air	Dampness indices
Deng et al. (79)	Indoor	Postnatal exposure to indoor dampness	Asthma incidence
Zheng et al. (80)	Indoor	Home environmental factors (dampness)	Childhood pneumonia
Zhang et al. (81)	Indoor	Home environmental factors (dampness)	Asthma and rhinitis
Hu et al. (82)	Indoor	High levels of indoor PM2.5 and VOCs (in house dust)	Children's health
Liu et al. (54) and Deng et al. (77)	Indoor	Indoor air pollution, renovation, use of gas for cooking, keeping pets, and living with smokers	Human health
Yu et al. (83)	Indoor	Solid fuels combusted	Cardiovascular mortality

In terms of the outdoor environment, epidemiological researchers have found that ambient air temperature changes are linked to spikes in cardiovascular disease morbidity (50–52) and death (6, 84). Previously researchers suggested that every 1°C increase/decrease in temperature above/below-specified points of reference increases the risk of heart disease (85, 86). Several researchers used maximum and minimum standard temperature ranges to investigate the health impacts of severe high and low temperatures (63). Luo et al. (87) also found that the relationships between PM2.5 and cardiovascular morbidity were more responsive to different temperature and relative humidity modifications. There is significant proof that there is a relationship between surrounding temperature and the growth of respiratory disorders such as COPD, bronchitis, upper respiratory infection, and asthma. The increased mortality of respiratory symptoms is linked to a susceptibility to unfavorable ambient air temperatures (53, 88). The combined impacts of meteorological variables may have an impact on respiratory illnesses. For example, PM2.5 and PM10 can lead to 17.30 and 14.72% of total COPD hospitalizations, respectively, on days with surprisingly low air temperatures (89). Environmental variables have also been connected to the occurrence of gastrointestinal system disorders (56), endocrine and metabolic mortality (57), hemorrhagic stroke, kidney pain, diabetic fatalities (58), and renal syndrome (59). The temperature has been demonstrated to substantially impact gastrointestinal system illnesses in several investigations. Infectious illnesses have been associated with environmental variables in studies (60, 61, 63). Dengue fever has increased by 11.9 and 9.9% for every 1°C rise in the highest and lowest air temperatures. Humidity levels reaching 78.9% were adversely related to dengue fever probability (90).

Home air quality is connected to outside environmental quality to some extent. People in cities devote around 90% of their time indoors (91). As a result, the indoor environment has a significant impact on human health. The physical parameters of indoor air temperature and humidity are linked to the concentrations of outside air contaminants. Variations in indoor temperature and relative humidity are likely to produce headaches, chest tightness, dry skin, and other symptoms in an indoor weather pattern. The influence of interior development on the environment has received significantly less attention than the influence of the outer environment. As a result, we choose the indoor and outdoor quality of the environment that may affect public health.

Most of the researcher's influence on indoor activities on the environment has taken place in large cities. A thorough analysis indicated that access indoors lower ambient temperature and humidity levels in the winter, and moisture in the summer might impact people's health (73). The wetness indices were favorable to dry and humid air (76). High levels of indoor PM2.5 and volatile organic compounds (VOCs) in-home dust were identified as potential health risks for youngsters (82). Indoor remodeling, use of gasoline for cooking, keeping pets, and living with people who smoke all influence public health and Indoor climatic elements and air quality (77). Yu et al. (83) also found that burning solid fuels inside increases the risk of cardiovascular death. Solid fuels emit various airborne pollutants, primarily particulate matter (PM).

Ambient temperature, moisture, and humidity are important elements for human and animal health in the indoor environment, specifically for the sensitive group (i.e., children). For example, children's lung and respiratory issues are caused by indoor air quality (i.e., temperature ranges and moisture content) (73). Respiratory problems are caused by household air pollution (19, 92). Allergies and asthma in children are caused by indoor mold and humidity (76); and indoor exposure to certain other environmental factors such as restoration work, preparing food, owning animals cause serious diseases to public health (77, 78).

## Data and Methods

This study provides a detailed review of human health, environmental quality, and governance quality. In addition, the study conducts an empirical analysis using the annual data of low-income countries from 1996 to 2020. The study reports novel solutions regarding the influence of economic, non-economic, and governance indicators on human health. Therefore, according to the existing literature, this study adopts six variables' data. This study uses the Incidence of Malaria (MI) as a proxy for human health. However, the explanatory variables include greenhouse gas emissions captured by metric tons of CO_2._ For economic performance, we use the economic growth factor indicated via gross domestic growth (GDP), governance or institutional quality is captured by regulatory quality (RQ), and government effectiveness. Besides, Domestic general government health expenditures (GHE) are also examined in the empirical model (18). Data for these variables are obtained from multiple sources, covering the 1996Q1 to 2020Q4 period for low-income countries^1^. The primary reason for adopting the small sample is the unavailability of data, where the available data on the World Bank site is only for 24 years. The variables and data specifications are reported in Table 3.

**Table 3:** Variables specifications.

**Variable**	**Specification**	**Data source**
*MI*	Incidence of Malaria (per 1,000 population at risk)	http://apps.who.int/ghodata/
*GHG*	Total greenhouse gas emissions (thousand metric tons of CO_2_ equivalent excluding Land-Use Change and Forestry).	https://www.climatewatch-data.org/ghg-emissions
*GDP*	Economic progress as GDP (constant 2015 US$)	https://databank.world-bank.org/source/world-development-indicators
*GE*	Government Effectiveness as governance-related measure of countries' performance	https://databank.world-bank.org/source/worldwide-governance-indicators
*RQ*	Regulatory quality on government performance. The estimate is the country's score on the aggregate indicator, ranging from −2.5 to 2.5.	https://databank.world-bank.org/source/worldwide-governance-indicators
*GHE*	Domestic general government health expenditure (% of GDP)	http://apps.who.int/nha/-database

Following the recent study of Farooq et al. (93), this study constructed the following empirical model.


(1) $$\begin{array}{r} MI_{i}=f\left(GHG_{i},GDP_{i},RQ_{i},GHE_{i},GE_{i}\right) \end{array}$$

The empirical models demonstrate that *GHG*_*i*_, *GDP*_*i*_, *RQ*_*i*_, *GHE*_*i*_, and *GE*_*i*_ are the functions of *MI*_*i*_. However, the empirical models adopt the following econometric form for empirical examination:


(2) $$\begin{array}{l} MI_{i}=\gamma_{0}+\gamma_{1}GHG_{i}+\gamma_{2}GDP_{i}+\gamma_{3}RQ_{i}+\gamma_{4}GHE_{i}\\ \;+\gamma_{5}GE_{i}+\varepsilon_{it} \end{array}$$

Where γ′*s* are the coefficients to be estimated and γ_0_ is the intercept in both the equations. Whereas *I* is the subscript that shows the cross-section of countries. Besides, the “ε” is the random error term of the regression model. It is important to mention that low-income countries' data is used as time-series data. For empirical analysis, the study used unit root testing, FMOLS, DOLS, and CCR regressions.

## Empirical Results

### Descriptive Statistics

As the first step of the analysis, the descriptive statistics of the research show the average values of gross domestic product (GDP), malaria incidence (MI), greenhouse gas emissions (GHG), government effectiveness (GE), government health expenditure (GHE), and regulatory quality (RQ). The descriptive statistics include mean, maximum, and minimum values, reported in Table 4. The minimum values are somehow nearby to their median values depicting the balancing point of the data. The standard deviation represents the volatility of the data and how much data is spread around its mean values. The overall values of the data describe that the data is symmetrical and normally distributed. GDP and GHG are steady and have almost same values in median, minimum and maximum parameters, while GE and RQ values are nearby in median, minimum and maximum parameters.

**Table 4:** Descriptive statistics.

**Variable**	**Obs**	**Mean**	**Std. dev**.	**Min**	**Max**
*MI*	96	240.23	34.3470	190.42	285.21
*GHG*	96	13.714	0.16364	13.4388	13.918
*GDP*	96	26.524	0.27830	26.044	26.896
*GE*	96	19.185	1.61203	17.26	22
*RQ*	96	19.781	1.84953	17.4	23.4
*GHE*	96	28.987	11.7715	13.696	44.5919

### Unit Root Tests

After the descriptive analysis of the data, the stationarity tests results of the data in the time series are shown in Table 5. Unit root tests are applied to determine the stationarity properties of data. Usually, the non-stationary trendy data depicts some association in the long run. Additionally, the unit root is the pre-testing test for cointegration analysis in the econometric time-series study. The Augmented Dickey-Fuller test is applied for testing unit root for the residuals. The test findings depict that the data is stationarity at first difference. Gross domestic product and government health expenditure are stationary at a one percent level of significance, greenhouse gas emissions, government effectiveness, and regulatory quality are significant at a five percent level of significance, whereas only malaria incidence is significant at a ten percent level of significance. The coefficients of the first difference are negative but statistically significant. The more the negative value depicts a stronger unit root, the more the null hypothesis will be rejected.

**Table 5:** Unit root testing.

**Variables**	**Augmented Dickey-Fuller**	
	**I(0)**	**I(1)**
*MI*	−1.6100	−1.6122*
*GHG*	1.248	−2.2241**
*GDP*	−1.546	−4.551***
*GE*	0.1612	−1.661**
*RQ*	1.764	−2.5431**
*GHE*	−0.0076	−4.431***

Significance is indicated by 10, 5, and 1% through *, **, and ***. I(0) is for level, and I(1) is for the first difference.

### Long-Run Estimates

We also analyze the empirical model test results of the data in Table 6. The article aims to look at the effects of government policies and environmental problems on people's health. The previous findings indicated that the variables in the research project are cointegrated. Both models' long-run estimations are based on three cointegration estimates: fully modified ordinary least squares (FMOLS), dynamic ordinary least squares (DOLS), and canonical cointegrating regression (see Table 6) (CCR). Other econometric estimators do not perform as well as these estimators. We use malaria infections as a dependent variable with other variables. Table 6 represents the empirical outcomes of these regressions.

**Table 6:** Empirical results of empirical model.

**Dep. Var. *MI***	**Coefficients**		
	**[Std. Error]**		
	**FMOLS**	**DOLS**	**CCR**
*GHG*	0.314*** [0.0361]	0.361*** [0.0462]	0.345*** [0.0321]
*GDP*	−0.033*** [0.0031]	−0.033*** [0.0043]	−0.030*** [0.0034]
*GE*	0.037*** [0.0038]	0.035*** [0.0037]	0.024*** [0.0021]
*RQ*	0.0044 [0.0032]	0.0011 [0.0014]	0.0010 [0.0040]
*GHE*	0.0421** [0.0048]	0.0411** [0.0037]	0.034* [0.0031]
*Constant*	29.693*** [0.2319]	26.897*** [0.2637]	28.984*** [0.2379]

Significance is indicated by 10, 5, and 1% through *, **, and ***. The standard error is provided in the brackets.

The coefficient values of gross domestic product (GDP), greenhouse gas emissions (GHG), and government effectiveness (GE), variables in FMOLS, DOLS, and CCR are statistically significant at a one percent level of significance, while coefficient values of government health expenditure variable in FMOLS, DOLS and CCR are statistically significant at a five percent level of significance. All other variables in long-run estimates are positive and statistically significant except for regulatory quality, which is statistically non-significant. The findings of the first model depict that greenhouse gas emissions (GHG), government health expenditure (GHE), and government effectiveness (GE) positively influence malaria incidence (MI). A percentage increase in these variables will improve the malaria incidence. Likewise, gross domestic product (GDP) has a negative but significant impact in the long run. The results are similar across the three estimators. The outcomes are identical across all three cointegration estimates.

## Discussion on Climate Change, Governance, and Public Health

### Discussion

The likelihood of high-temperature death and disease has increased due to global warming. According to the International Panel on Climate Change (IPCC), other health outcomes have been influenced, including those resulting from a decrease in food availability to youngsters (94, 95). Overall, the existing literature supports our empirical findings, and these are justified. For instance, the governance quality in low-income countries is relatively poor, which leads to social issues and health-related problems. Two primary techniques are necessary to safeguard public health: mitigation, or large reductions in carbon dioxide emissions, which corresponds to prevention and treatment; and adaptation, or activities to anticipate and lessen threats, which relates to treatment and prevention (or public health preparedness). There are several ways of dealing with the problem of global warming, a variety of options are available. Many of these would instantly enhance one's health. Several viable remedies for environmental catastrophes and health hazards range from lowering chronic illness rates to decreasing motor vehicle collisions. Reducing greenhouse gas emissions, implementing sustainable energy technology, altering transportation patterns, and improving building design are all doable, expensive, and appealing to many stakeholders. Health practitioners are uniquely positioned to develop policies that benefit the environment and people (96). Climate change can increase numerous environmental health concerns that doctors and public health experts are acquainted with (97). Risks and population susceptibility will differ by area; indirect implications, such as ecological collapse, may eclipse more direct health impacts, which are more difficult to assess.

According to the IPCC, climate changes have become more common because of global warming. More research suggests that unusual climatic circumstances may enhance the risk of epidemiological mortality and morbidity in global climate change. According to a study published by the World Health Organization, 12.6 million people (including 2.987 million in China) died because of bad working and living conditions, accounting for 23% of all deaths (14). Risk factors that have been identified, such as harsh weather and air quality, have been proposed to harm human health and cause mortality and morbidity (98). Several observational types of research in China (52, 99) and other countries (100) highlighted the effects of environmental temperature, multiple data sources, and actual temperature on public health. Severe global temperatures have a major influence on human health, according to conclusive evidence (53), particularly within vulnerable communities (e.g., the elderly, children, and persons with chronic conditions) (101). Seasonal temperature fluctuations also greatly impact human population health, such as death rates from heart, lung, and other infectious disorders (102).

States are being affected by global warming, and they rapidly realize the need to plan for the effects on their infrastructure and citizens. Several cities have observed significant increases in the frequency and severity of extreme weather events; others have seen changes in temperatures; and still, others have seen coastline erosion, wetlands disappearing, and storm surges (103). During the planning and organizing phase, stakeholders must understand what to expect to build the required structures (104). Adaptation and planning techniques are being developed in several cities. Natural ecosystems perform a variety of mitigating activities in this regard. Even though it is necessary to discuss a methodology for and understanding of various types of terrains, as well as a theoretical background, the most important thing is to establish a common set of standards that is compatible with the different ways in which disciplines and community members perceive and value landscapes (105).

**Figure 2:**
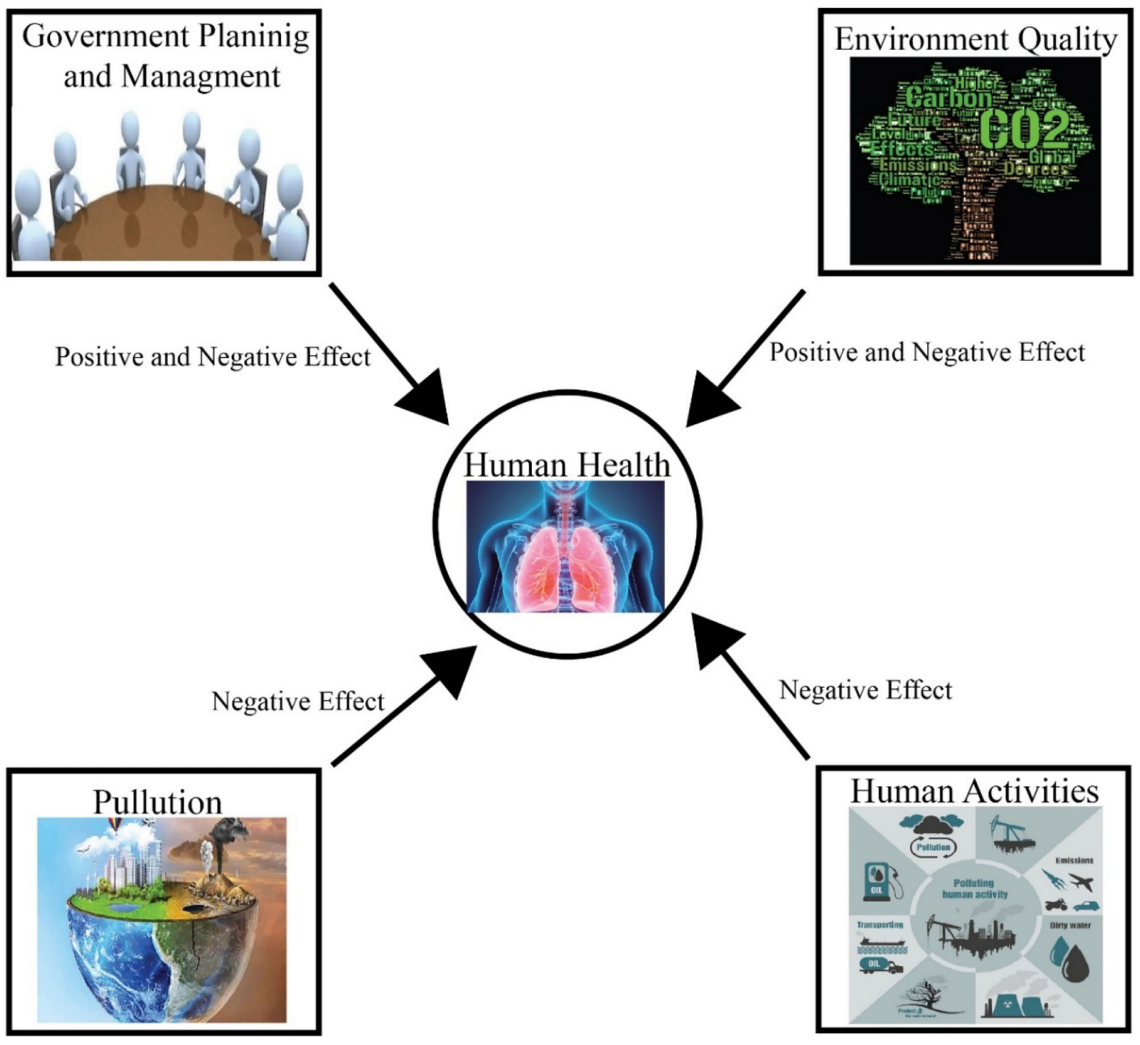
Main factors that cause good and bad effects on human health.

Climate change, pollution, human activity, and government policies all have a variety of positive and negative consequences on public health (Figure 2). Climate change has been related to bad health outcomes for the past 20 years, hence actions to reduce global warming and the accompanying negative repercussions are necessary. Although climate changes may have substantial ramifications for the health of the world's people, high-quality research and responsible, educated dialogue must continue (106, 107). Understanding the link between global warming and health and establishing strategies to secure a sustainable future while safeguarding health will formulate effective future policies. Besides dealing with climate change, co-benefits can offer policymakers additional incentives to cut carbon dioxide emissions and short-lived climatic pollutants. Accounting for benefits may show that lowering greenhouse gas emissions produces net economic gains (66), as well as increased labor productivity (108), and reduces health system costs (109).

### How to Improve the Environment Footprint?

The environment is essential to human survival and growth. People's physical, mental, and social health are all influenced by their surroundings. Despite great advances, major differences in environmental quality and public health exist within and within European countries. As the economy has evolved, contradictions between human and natural surroundings (e.g., air, ocean, forest, and water) have grown more obvious. Environmental variables and human health should be evaluated in a wider geographical, social, and cultural context, considering various routes and interactions. Environmental pollution governance can help with present environmental difficulties and the economic challenges different nations face. A robust foundation for economic growth and pollution management's integrated development is a prerequisite for long-term sustainability.

International trade (import/export trade), according to some studies, can help impoverished countries improve their environmental quality, hence improving global environmental quality (110). According to some studies, international trade has impacted the ecology of developing nations. According to Ang (111) and Dean et al. (112) developing nations, such as China, have reduced environmental liabilities in global trade, resulting in increased local pollution and transforming these countries into pollution havens for polluting corporations from developed countries. Cai et al. (113) came to a consistent conclusion on the pollution haven's effect during their study.

## Concluding Remarks and Approaches to Climate Change Adaptation

Because of the critical role of governance in the health community, effective governance has been a hot issue in public sector management in recent years. According to studies and data worldwide, “good governance” is one of the most critical characteristics in emerging countries. The cornerstone of progress is so-called good governance. The impact mechanism of government environmental regulation, public engagement, and their coordinated influence on public health are examined in this study. The study used the data of low-income countries from 1996Q1 to 2020Q4 and reported interesting empirical findings. For empirical analysis, the study used unit root testing, FMOLS, and DOLS regressions. The empirical findings reported few interesting findings. An important finding of this paper revealed that governance quality and environmental pollution in low-income countries is the main culprit for health issues. It shows a more than the urgent need for policymakers and government institutions to design novel policies and reforms for mitigating pollutant emissions and improving the governance quality. Above that, the results can motivate the government to enhance the environmental, governmental system, which benefits for global health.

The best adaptation techniques accomplish many goals. Improvements to key infrastructure might aid global warming adaptation. Vegetation, building siting, white roofs, and architectural design, for example, can help to lessen the urban heat island effect and climate control power consumption. According to recent research, air conditioning waste heat may warm outdoor air by more than 1°C, reducing the requirement for air conditioning strongly impacts urban heat islands (114).

1. Green spaces such as woodlands and parks not only help to minimize heat islands but also help to reduce stress (115–117), neighborhood social cohesiveness (118), and crime and violence (119, 120). Rather than a single technological solution, an ecosystem perspective can provide several advantages and efficiency improvements (121).
2. Seawalls have often been used to maintain shorelines when the sea level rises. This coastal habitat also protects local fisheries by preserving wetlands and marine feeding chains. Growing mangroves for severe storm protection in Vietnam, on the other hand, costs one-seventh of the cost of developing and maintaining seawalls or dikes for the same reason (122).
3. Lack of physical activity is a major risk for various non-communicable illnesses and is thought to cause 3.2 million deaths per year (123). Increased urban walking and cycling, sometimes known as “active transport,” has significant health advantages. This strategy may deliver the greatest immediate benefits by reducing health-damaging pollution levels and enhancing fitness.
4. In high-consumption populations, reduced meat intake has health advantages. Agriculture, livestock production, and forestry account for around 24% of global greenhouse gas emissions (94), with meat and dairy accounting for most pollutants.
5. Increasing wind, solar, wave, and geothermal heat have health and climatic advantages. According to Wisconsin research, enhanced electrical power efficiency and renewable production, which minimize carbon emissions at a low cost, might lower statewide nitrogen oxide emissions by 55% and sulfur dioxide emissions by 59 percent (124).
6. The cost of sustainable energy transition and transport network reorganization programs continues to be a cause of worry. The National Health Service in England and Wales would save $25 billion over 20 years if active transportation managed to reach Copenhagen levels (109); in the United States, $3.8 billion per year (95 % CI, $2.7–$5.0 billion) would be saved through physical training advantages result of increased biking (125). Increased opportunities for physical fitness are likely to result in further financial benefits.

Every approach to reducing carbon emissions should provide a risk assessment to ensure that potential benefits and dangers are considered in estimated costs and unintended effects. Government policies are crucial in recognizing and communicating the health dangers of climate change and the benefits of using fewer fossil fuels. Health professionals play a unique role in policy concerns such as energy, housing, transportation, urban planning, agriculture, food systems, and more. Environmental awareness needs to be promoted, pollution needs to be handled, and future environmental quality needs to be controlled.

## Data Availability Statement

The original contributions presented in the study are included in the article/supplementary material, further inquiries can be directed to the corresponding author/s.

## Author Contributions

HC and LZ: conceptualization and writing—original draft preparation. HC: methodology, supervision, and funding acquisition. LZ: software. LZ, YL, and HC: validation. YY: formal analysis, resources, and project administration. YL: investigation. YY, LZ, and HC: data curation. HC and YY: writing—review and editing. YL and LZ: visualization. All authors have read and agreed to the published version of the manuscript.

## Funding

This research was funded by the National Social Science Fund General Project of China (No. 19BGL092), Innovation Strategy Research Project of Fujian Province (No. 2021R0156), GF Securities Social Welfare Foundation Teaching and Research Fund for National Finance and Mesoeconomics.

## Conflict of Interest

The authors declare that the research was conducted in the absence of any commercial or financial relationships that could be construed as a potential conflict of interest.

## Publisher's Note

All claims expressed in this article are solely those of the authors and do not necessarily represent those of their affiliated organizations, or those of the publisher, the editors and the reviewers. Any product that may be evaluated in this article, or claim that may be made by its manufacturer, is not guaranteed or endorsed by the publisher.

## Footnotes

^1^ https://data.worldbank.org/country/XM
